# Genomics of Trypanosomatidae: Where We Stand and What Needs to Be Done?

**DOI:** 10.3390/pathogens10091124

**Published:** 2021-09-02

**Authors:** Vyacheslav Yurchenko, Anzhelika Butenko, Alexei Y. Kostygov

**Affiliations:** 1 Life Science Research Centre, Faculty of Science, University of Ostrava, 710 00 Ostrava, Czech Republic; anzhelika.butenko@paru.cas.cz; 2 Martsinovsky Institute of Medical Parasitology, Tropical and Vector Borne Diseases, Sechenov University, 119435 Moscow, Russia; 3 Institute of Parasitology, Biology Centre, Czech Academy of Sciences, 370 05 České Budějovice, Czech Republic; 4 Zoological Institute of the Russian Academy of Sciences, 190121 St. Petersburg, Russia

**Keywords:** trypanosomatids, next-generation sequencing, genomics

## Abstract

Trypanosomatids are easy to cultivate and they are (in many cases) amenable to genetic manipulation. Genome sequencing has become a standard tool routinely used in the study of these flagellates. In this review, we summarize the current state of the field and our vision of what needs to be done in order to achieve a more comprehensive picture of trypanosomatid evolution. This will also help to illuminate the lineage-specific proteins and pathways, which can be used as potential targets in treating diseases caused by these parasites.

## 1. Introduction

The flagellates of the family Trypanosomatidae represent one of the most evolutionarily successful groups of parasitic protists, adapted to an extremely wide range of hosts—from various animals (mainly insects and vertebrates) to flowering plants and even ciliates. Depending on whether their life cycle includes a single host or there is an obligate alternation between two different hosts, trypanosomatids are subdivided into monoxenous (predominantly insect parasites) and dixenous (typically insect-transmitted parasites of vertebrates or plants) [1]. Most research efforts have been focused on studying dixenous trypanosomatids of the genera *Trypanosoma* and *Leishmania*, which cause severe (often fatal) diseases in humans and domestic animals. Therefore, sequencing of trypanosomatid genomes started from the three important human pathogens: *Trypanosoma brucei*, *T. cruzi*, and *Leishmania major* [2,3,4]. A comparative study has shown that despite differences in genome size and gene content, these species share a relatively high level of gene order conservation (synteny) and overall genomic organization: most protein-coding genes are intron-less and form conserved polycistronic gene clusters, whereas species-specific genes predominate sub-telomeric or internal non-syntenic chromosomal regions [5]. The subsequent genomic studies expectedly focused on other species of these two genera with the clear preference for *Leishmania*, since it contains more species infective to humans. At the time of writing this review, the assembled genome sequences for multiple isolates of 24 species of *Leishmania* and about a dozen species and subspecies of the genus *Trypanosoma* are available in public databases (Table S1).

**Figure 1:**
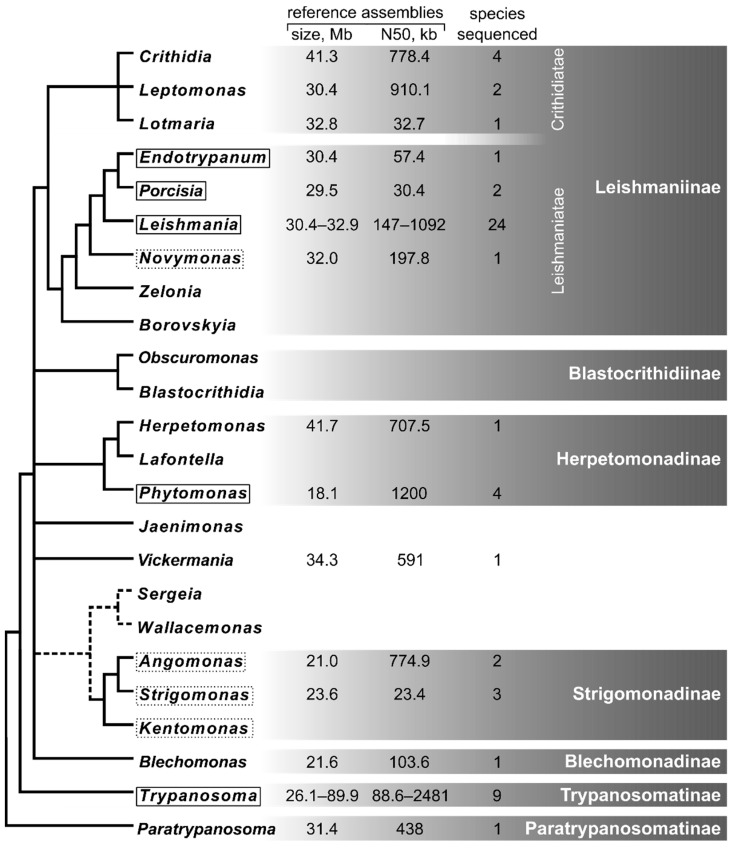
Schematic phylogenetic tree of Trypanosomatidae with a summary on sequenced genomes. Solid and dashed boxes mark dixenous and endosymbiont-bearing genera, respectively.

However, the diversity of trypanosomatids is predominantly represented by monoxenous parasites, from which their dixenous kin have originated at least three times independently [1]. These cases are *Leishmania* (along with *Endotrypanum* and *Porcisia*) spp. within subfamily Leishmaniinae, *Phytomonas* spp. in the subfamily Herpetomonadinae, and *Trypanosoma* spp. constituting a separate early-diverging lineage (Figure 1). The research interest in the monoxenous trypanosomatids has significantly increased in the last decade; of note, 12 out of the 19 currently recognized genera of these flagellates have been described within this short period [1].

The studies of insect-dwelling flagellates are important for better understanding not only the biology of their dixenous relatives, but also eukaryotic evolution in general [6]. For example, the members of the genus *Blastocrithidia* evolved an idiosyncratic genetic code with all three stop codons used for coding amino acids [7]. Some trypanosomatids, namely *Novymonas* and the three genera of the subfamily Strigomonadinae (*Angomonas*, *Strigomonas*, and *Kentomonas*) harbor intracellular bacterial symbionts [8,9,10]. These endosymbionts complement the metabolic requirements of their flagellate hosts with pathways responsible for the synthesis of amino acids, vitamins, and heme [11,12,13,14]. The unusual genus *Vickermania* became biflagellate by disrupting the processes of cell division and flagellum duplication to resist the fly midgut peristaltic flow in the absence of an opportunity to attach to the intestinal wall [15]. Various monoxenous trypanosomatids independently acquired thermotolerance, a prerequisite of the transition to dixeny, and some of them have even been documented in vertebrates [16,17,18]. Below, we review the current state of genomic research in trypanosomatids with a focus on monoxenous species. The taxonomy is presented in accordance with [1].

## 2. *Trypanosoma* spp.

The first trypanosome, whose genome had been sequenced and analyzed, was the agent of African animal trypanosomiasis—*T. brucei brucei* [3,19] (Table S1). The studies of human-infective *T. b. gambiense* and *T. b. rhodesiense* demonstrated extremely high similarity of the genomes in all three subspecies, conservation of the variant surface glycoprotein (VSG) repertoire, and only rare segmental duplications [20,21]. In *T. b. evansi*, mechanically transmitted by insects and lacking kinetoplast, the procyclin-associated genes needed for the development in the vector have been lost or disrupted, and the γ-subunit of ATP synthase, which is involved in generation of the mitochondrial membrane potential in the absence of kDNA, has mutated [22,23]. The comparison of the genomes of all the above subspecies did not allow identification of factors leading to pathogenicity in humans. Two draft genome assemblies of *T. b. equiperdum*, which is dyskinetoplastic (lacks part of its kDNA) due to the loss of the vector part of its life cycle, have been published with no accompanied analysis [24,25]. Several studies of the genome of the tsetse-transmitted *T. congolense* focused on the analysis of its VSG repertoire and its comparison to that of *T. brucei* [26,27,28,29]. They revealed several important differences in the organization and functioning of the VSG expression sites, including the absence of conserved repeats flanking the VSG loci and the scarcity of expression site associated genes in *T. congolense*, and the scale of recombination. *Trypanosoma vivax* genome encodes the most diverse VSG repertoire among all investigated trypanosomes [26,30].

The studies of the *T. cruzi* genome involved numerous strains of this species, allowing to improve the quality of the existing assemblies and providing a deeper insight into its population structure [31,32,33,34,35,36,37,38,39,40,41]. A recent genome analysis of two *T. cruzi* strains revealed that the rapid evolution of gene families involved in immune evasion is one of the major contributors to the intraspecific genome variation in this species [42]. Interestingly, despite the shorter overall length, multiple genes were acquired by lateral gene transfer and some gene families underwent expansions in the genome of a bat-infecting species *T. marinkellei*, which is closely related to *T. cruzi* [43]. Genomes of human non-pathogenic *T. rangeli* and the bat parasite *T. conorhini*, representing a clade related to that of *T. cruzi*, have less retrotransposons and multigene family copies, but more genes involved in the biosynthesis of carbohydrates [44,45]. The crocodile-infecting species *T. grayi* was shown to lack surface proteins (mucins and VSGs), which are characteristic for other trypanosomes investigated thus far [46]. The genome analysis of ruminant-parasitizing *T. theileri* revealed several new families of surface proteins, as well as a general conservation of core cellular metabolic pathways [47].

What needs to be done: The genus *Trypanosoma* corresponds rather to a subfamily than to a single genus—it is very speciose (over 500 described species) and diverse. According to the latest taxonomical revision, it includes sixteen subgenera and several undescribed lineages of the same level [1]. Only a few of these have been analyzed to date, and this significantly limits our understanding of the evolution of parasitism in this group (Table S1). Surprisingly, the genome of one of the most common trypanosome species, flea-transmitted *T. lewisi*, which typically inhabits rats [48], but occasionally infects humans [49], has not been analyzed yet. Of special interest would be the genomic analyses of anuran trypanosomes (subgenus *Trypanosoma*), which gave rise to the parasites of fish and may represent the ancestral group for all terrestrial subgenera [50]. The representatives of this subgenus are expected to keep archaic traits of genomic organization, inherent to the common ancestor of trypanosomes, and their study using NGS might shed light on the origin and evolution of some important gene families, such as VSGs, procyclins, mucins, etc.

## 3. Dixenous Leishmaniinae

Out of the four *Leishmania* subgenera, i.e., *Leishmania*, *Mundinia*, *Sauroleishmania*, and *Viannia*, early genomic studies have focused on the first one (in particular, *L. major*, *L. donovani*, *L. infantum*, *L. mexicana*), and only *L.* (*V.*) *braziliensis* was used for comparison. Those studies revealed extremely high synteny levels, interspecific differences in the gene content, and associations of some genes with drug resistance phenotype [2,51,52] (Table S1). More *L*. (*Leishmania*) species and strains were analyzed later [53,54,55,56,57,58,59,60,61,62,63].

Later on, the subgenus *Viannia* started to receive more attention. Comparative genomic analysis of *L. braziliensis* and *L. peruviana* demonstrated substantial differences in gene content, chromosome copy number, as well as numerous SNPs and indels [64,65,66]. Sequencing of *L*. *panamensis* genome uncovered several mobile elements absent from the genomes of *L*. (*Leishmania*), along with a higher number of pseudogenes compared to the latter [67]. The study of *L. naiffi* and *L. guyanensis* genomes identified common features of the subgenus *Viannia*, such as aneuploidy, the presence of about 20 subgenus-specific gene families, and a high content of TATE transposons [68,69].

The early genomic study of a lizard parasite *L.* (*Sauroleishmania*) *tarentolae* demonstrated the loss of genes involved in oxidative stress protection and vesicular-mediated protein transport, as well as those expressed in *L*. (*Leishmania*) amastigotes. Meanwhile, the surface glycoprotein GP63 and promastigote surface antigen PSA31C gene families are expanded in this species [70,71]. Other studies of a species from this subgenus—*L. adleri* infecting rodents and lizards—has identified gene amplification, changes in chromosome copy number, and chromosome fission events [72,73].

The genome assemblies of *L*. (*Mundinia*) spp. were found to be similar in size to those of *Sauroleishmania*, but smaller than those of *Leishmania* and *Viannia*, due to multiple gene losses and gene family contractions [74]. The absence or reduction in the number of lipophosphoglycan-modifying side chain galactosyltransferases and arabinosyltransferases, as well as β-amastins has confirmed previous reports on the differences in cell surface architecture in *L.* (*Mundinia*) and other *Leishmania* spp. [75,76,77].

*Endotrypanum monterogeii* and *Porcisia* spp., being dixenous parasites of sloths and porcupines, respectively, represent the closest known relatives of *Leishmania.* The recently published analysis of their genomic sequences shed light on the evolution of pathogenicity in dixenous Leishmaniinae, which appears to be shaped mainly by changes in the amastin repertoire [78].

*L. donovani* and *L. braziliensis*, are the only trypanosomatids, to which single-cell genome sequencing approach has been applied thus far [79]. While the respective methods are widely used in human and cancer research, their application is restricted to just a handful of pathogenic species, including some apicomplexans and *Leishmania* [80]. Single-cell genome sequencing is instrumental in investigation of the haplotype diversity and de novo mutations in populations of pathogens. It allowed to characterize the karyotypes of *L. braziliensis* cells demonstrating mosaic aneuploidy [79]. A combination of multiple types of omics data originating from single trypanosomatid cells will provide a holistic view on the interactions of these pathogens with their hosts.

What needs to be done: The genus *Leishmania* is not as speciose as *Trypanosoma*, and the genomes for most of its representatives have been already sequenced with the exception of the poorly studied subgenus *Sauroleishmania*, for which 19 species have been described [81]. The peculiarities of the life cycles of these lizard-dwelling flagellates, such as their presence in the host gut and ability to infect a wide range of the mononuclear cells, erythrocytes, and thrombocytes [82,83], warrant further studies. Meanwhile, only one species has been analyzed for the genus *Endotrypanum*—*E. monterogeii*, and adding at least *E. colombiensis* (previously classified into *Leishmania* [84]), which can infect humans, would be important for understanding the pathogenesis of these flagellates. In addition, several genome assemblies of *Leishmania* spp. are available in public databases waiting to be analyzed and put into the context of comparative studies [85,86,87,88].

## 4. Monoxenous Leishmaniinae

Genomes for several monoxenous representatives of the subfamily Leishmaniinae have been sequenced and analyzed. The study of *Lotmaria passim*, *Crithidia bombi*, and *C. expoeki*, parasitizing agriculturally important Hymenoptera (honeybees and bumblebees), demonstrated numerous examples of horizontal gene transfer [89,90]. Genomic analysis of the latter two species at the population level has revealed that different strains vary considerably in terms of single nucleotide polymorphisms and gene copy number with a pattern fitting a scenario of rapid host-parasite coevolution, where the selective advantage of a given parasite strain is only temporary [91]. The genome and transcriptome sequencing of *Leptomonas seymouri*, the species repeatedly found in clinical samples along with *Leishmania donovani* [92], has allowed identifying its pre-adaptations to dixeny [17]. The genomic data of *Leptomonas pyrrhocoris*, an omnipresent parasite of firebugs, which has been proposed as a new model trypanosomatid species, were used to find new virulence factors of *Leishmania* [93]. The transcriptomic study of *Crithidia thermophila* showed a clear distinction in the mechanisms of thermotolerance in this species and *L. seymouri* [16]. The *C. fasciculata* RNA-seq data were used to elucidate potential mechanisms for insect-specific adhesion in trypanosomatids [94]. The available genomic data of *C. acanthocephali* made possible the comparative analysis of the endosymbiont-bearing and aposymbiotic species [14]. Two species closely related to *C. fasciculata* have been recently reported from human infections and their genomes have been sequenced [18,95]. The genome of the endosymbiont-bearing *Novymonas esmeraldas*, the closest known relative of dixenous Leishmaniinae, revealed a very similar gene content to the latter with the large number of GP63 proteases and pteridin/biopterin transporters, recognized virulence factors of *Leishmania* spp. Owing to the presence of the endosymbiont, this flagellate became prototrophic for all amino acids, heme, and most vitamins, i.e., even more independent of the presence of essential nutrients in the host than Strigomonadinae [12,96].

What needs to be done: Sequencing of additional species belonging to the non-monophyletic genera *Crithidia* and *Leptomonas* will help to delineate the entangled taxonomy of the infrafamily Crithidiatae (Figure 1). In addition, this lineage presents good examples of species with narrow and broad host specificity, which would be interesting to compare from the genomic point of view (e.g., *L. pyrrhocoris* is restricted to firebugs [97], while various species of true bugs and flies are documented for *C. brevicula* [98,99]). Although *Novymonas* is the closest relative of dixenous Leishmaniinae, the acquisition of endosymbionts resulted in very specific adaptations. Therefore, sequencing the genomes of other monoxenous trypanosomatids of the infrafamily Leishmaniatae (genera *Zelonia* [84] and *Borovskyia* [100]) is needed to illuminate the evolutionary origin and molecular signatures of dixenous Leishmaniinae.

## 5. Herpetomonadinae

The less studied lineage Herpetomonadinae is another subfamily containing dixenous parasites (plant-dwelling *Phytomonas* spp.) along with their monoxenous relatives (Figure 1). Some of the latter appear to be on the way to dixeny, as judged by their detection in plants [101,102] or vertebrates [103]. The analysis of four available genomes of *Phytomonas* spp. (those are *Phytomonas* spp. (isolates EM1 and Hart1) [104], *P. serpens* (isolate 9T) [105], and *P. françai* [106]) revealed additional peculiarities of these plant-inhabiting flagellates, such as significant genome streamlining at the expense of intergenic regions, mobile elements and narrowed gene repertoires, as well as the absence of some electron transport chain proteins. The only *Herpetomonas* species whose genome has been sequenced to date is *H. muscarum* [14,107]. It was used as a reference for the comparative analyses either with endosymbiont-bearing or dixenous trypanosomatids, therefore it is not clear what are its own peculiarities.

What needs to be done: Of special interest would be genomic studies of the speciose genus *Herpetomonas*, which actively explores various ecological niches. Ancestrally, these flagellates are parasites of (brachyceran) flies, but some of them switched to parasitism in true bugs, cockroaches, mosquitoes, or biting midges, while one species, *H. samuelpessoai*, demonstrates an astonishing ecological plasticity and has been isolated also from plants and even a human patient [1]. These should shed light on the adaptation of trypanosomatids to different hosts and environments. Although the genomes of *Phytomonas* spp. have been already investigated, the analysis was restricted to only four species inhabiting the phloem, latex or fruit and representing the “crown” of this lineage. Thus, the genomic features observed in these flagellates represent a derived state and it is still not clear what has allowed these flagellates to become dixenous. Therefore, the genomes of some early-branching species, such as *P. lipae* and *P. oxycareni* infecting seeds [108,109] need to be analyzed and compared with those of the two closely related monoxenous genera *Herpetomonas* and *Lafontella* [110], which would serve as outgroups. Of special interest would be to study the genomes of the secondarily monoxenous *P. nordicus* [111] (to identify genomic features associated with dixeny in this genus) and its closely related species—*P. borealis*, possessing a bacterial endosymbiont, the relationship with which is likely distinct from those in *Novymonas* and Strigomonadinae [112].

## 6. Strigomonadinae

The genomes of endosymbiont-bearing Strigomonadinae and their intracellular bacteria (*Ca.* Kinetoplastibacterium spp.) have been studied quite intensively. A series of papers characterized the genomes of *Angomonas deanei*, *A. desouzai*, *Strigomonas oncopelti*, *S. galati*, and *S. culicis*, as well as the metabolic interactions with their symbiotic partners [13,14,113]. It was demonstrated that the amino acid biosynthetic pathways are interlaced between the endosymbionts and their flagellate hosts and that many genes had been acquired by Strigomonadinae from various groups of bacteria. The importance of Strigomonadinae led to the establishment of the first genetically-trackable system in the model species, *A. deanei* [114]. A recent study using genomic data of two *A. ambiguus* strains, *A. deanei*, and their endosymbionts demonstrated that bacteria from the latter species repeatedly replaced bacteria in the former [115].

What needs to be done: The genus *Kentomonas* represent the earliest branch within the subfamily [8] and, therefore, may keep in its genome some archaic traits inherent to the common ancestor of the subfamily. In addition, it has been shown to differ from its cousins in the dependence of external source of heme (or its precursors) [11] and may also diverge in other aspects of its metabolism. Hence, a genomic analysis of this trypanosomatid is warranted.

## 7. Other Monoxenous Lineages

There are three more monoxenous species, whose genomes have been sequenced and analyzed. Genome sequencing of the early-diverging *Paratrypanosoma confusum* and a representative of the flea-parasitizing genus *Blechomonas ayalai* has allowed to draw preliminary conclusions concerning the evolution of metabolic pathways in the family Trypanosomatidae [116,117]. The most recent addition to the collection of trypanosomatid genomes was that of *Vickermania ingenoplastis*, a species lacking mitochondrial respiratory complexes III and IV and, thus, mainly relying on glycolysis, similarly to *Phytomonas* spp. However, in contrast to the plant trypanosomatids, the genome of this flagellate did not shrink, but experienced a substantial expansion of some protein families, in particular, the glycolytic enzymes [118].

What needs to be done: Representatives of numerous trypanosomatid genera have not been sequenced and some of them have not even been studied since their original description. (1) *Blastocrithidia* and *Obscuromonas* of the subfamily Blastocrithidiinae (Figure 1) share a unique resistant developmental stage—the cyst-like amastigote [119]. Moreover, some of them demonstrate quite a complex development in insects, comparable to that in dixenous parasites [120,121]. It would be interesting to find the genomic basis of these peculiarities. (2) *Jaenimonas drosophilae* inhabits fruit flies and have been proposed as a model to study the insect immune response to trypanosomatid parasites [122]. Sequencing the genome of this parasite would ease using it as such and understanding its intimate relationships with the host. (3) The genus *Sergeia* parasitizes biting midges and sandflies [123] and thus represents a good model to study the challenges faced and solutions used by trypanosomatids in blood-sucking nematoceran Diptera. Importantly, the same host groups are used by medically relevant *Leishmania* spp. and, therefore, finding parallels in the genome evolution between them and *Sergeia* might provide additional information on the biology of the former [124]. (4) The symbiont-free genus *Wallacemonas* is closely related to Strigomonadinae (Figure 1) and is similar to them in morphology and lifestyle [119]. Thus, it represents a promising reference to reconstruct the metabolism of the ancestors of these endosymbiont-bearing flagellates and answers the question of why some trypanosomatids need endosymbionts, while others successfully live without them in the same hosts.

## 8. Other Applications of the Trypanosomatid Genomic Data

The availability of multiple representative genome sequences from various Trypanosomatidae enabled a robust analysis of the evolution of different gene families in this group. Some examples are provided below. The analysis of amastins, a large family of surface glycoproteins expressed primarily in amastigotes, revealed that δ-amastin subfamily is restricted to the dixenous Leishmaniinae and its expansion has likely happened in the ancestor of the genus *Leishmania* [78,125]. The repertoire of adenylate cyclases has expanded in dixenous trypanosomatids and many genes encoding these proteins pseudogenized in those subspecies of *T. brucei*, which lost the ability to develop in insects [126]. The analysis of myosin gene family suggested that these proteins were already diversified in the kinetoplastid common ancestor and secondarily, lost multiple times afterwards [127]. Genomic studies revealed that at least three trypanosomatid lineages—Leishmaniinae, Blastocrithidiinae, and *Vickermania*—independently acquired catalase from different groups of bacteria, whereas dixenous Leishmaniinae secondarily lost it [118,128,129]. The study of tubulin gene arrays demonstrated that while in the majority of trypanosomatid lineages and in the free-living bodonids that the α- and β-tubulin genes are alternated, in Leishmaniinae, these multicopy genes are organized in homogeneous (α-only and β-only) stretches [130]. The analysis of the evolution of trypanosomatid UDP-glycosyltransferases, the superfamily of enzymes participating in the modification of various surface macromolecules, showed their independent diversification in distinct groups of these parasites. Interestingly, one of the ancient lineages of these enzymes present in the free-living *Bodo saltans* has been lost from all trypanosomatids except stercorarian trypanosomes [131]. Side chain galactosyl and arabinosyltransferases of that large superfamily ensure lipophosphoglycan modifications needed for *Leishmania* attachment and detachment inside insects [132,133,134]. The analysis demonstrated differences in the repertoires of these enzymes between the subgenera *Leishmania* and *Viannia* correlating with the affinity of the flagellates to different intestinal sections of their different insect hosts [135,136]. In *Leptomonas pyrrhocoris*, which does not attach to the intestinal wall of its firebug host, the orthologs of these genes showed early divergence and expansion, suggesting distinct functions [137].

The analysis of gene families and comparative genomic studies discussed above can be hampered by the absence of contiguous assemblies with well-resolved repetitive regions. Although trypanosomatid genomes are relatively small (typically around 20–30 Mb), they contain many repeats and, therefore, it is challenging to obtain a chromosome-level assembly based on short sequencing reads [138]. For several trypanosomatid genera, more contiguous hybrid assemblies based on the combination of short and long sequencing reads have become available (Table S1). Application of a combination of long read sequencing and genome-wide chromosome conformation capture (Hi-C) enabled haplotype-specific assembly of *T. brucei* 427 Lister genome and revealed that antigen-encoding sub-telomeric regions are folded into distinct compact structures [139]. For *T. cruzi*, the trypanosomatid having the largest genome sequenced so far, a newer assembly obtained using Nanopore data, led to a significant increase of the number of identified single-copy orthologs and repetitive transposable elements as well as overall estimated genome size [37]. By far, the most contiguous genome assembly, which we suggest to use as a new reference for this species, was obtained recently using a combination of PacBio Single-Molecule Real-Time sequencing and proximity ligation methods [42]. One more example of a substantial quality improvement is the recently published Nanopore-based genome assembly for *Angomonas deanei*, which identified new chromosome-level features such as a supernumerary chromosome, a long inversion and a translocation [140]. After careful annotation of such genome assemblies based on multiple types of evidence (including transcriptomic and proteomic data), the trypanosomatid research community should consider using these new assemblies instead of the old references based solely on short reads and sometimes erroneous annotations.

Although no DNA viruses have been reported in trypanosomatids so far, the available genomic data for several *Leptomonas pyrrhocoris* strains has allowed identification of an endogenous viral element related to *Leppyr*TLV1 (a tombus-like single-stranded positive sense RNA virus), which was apparently captured via reverse transcription and integrated into the trypanosomatid genome [141].

Finally, next-generation sequencing data can be used for analyzing the composition and (to some extent) function of the kinetoplast. In this respect, the kinetoplast genomes of the two model species, dixenous *T. brucei* and monoxenous *Leptomonas pyrrhocoris*, have been scrutinized. Their analyses revealed novel non-canonical mechanisms, as well as species-specific differences in RNA editing [142,143]. Such studies can delineate not only the structure of maxicircles and minicircles [144,145,146,147], but also predict the guide RNA repertoire in a given species [143,148,149]. As judged from pre-genomic studies carried out on single genes, different lineages of trypanosomatids possess distinct kDNA editing patterns [150]. This, along with the abovementioned degradation of kDNA in two *T. brucei* subspecies, demonstrates the underestimated importance of the kinetoplast genome in the trypanosomatid development. Performing comparative studies on the editing using whole kinetoplast genomes with a wide range of trypanosomatid phylogroups should shed light on their particular life strategies and allow better understanding of the evolution of this fascinating group of parasites.

## 9. Conclusions

A fair number of trypanosomatid genomes have been sequenced and there is a significant progress in understanding their evolution, structure, and function. Nevertheless, many questions still remain unanswered and more of them arise, as new representatives of this group of flagellates are discovered and/or analyzed in broadscale biodiversity assays.

The relatively small size of trypanosomatid genomes makes these parasites an attractive model to study how the evolution of traits and genomes are correlated. This is further facilitated by the possibility to cultivate and genetically modify many trypanosomatids, combined with a knowledge of their diversity. However, as judged from the environmental screens (for example, refs. [151,152] and many others), there are still taxa of the generic level and above to be described. Meanwhile, the lack of data on the biology of many trypanosomatid groups still represents an important obstacle in interpreting the observed genomic differences, therefore, more data on trypanosomatid development, strategies of transmission, host-parasite interactions, etc., are needed.

## Supplementary Materials

The following are available online at https://www.mdpi.com/article/10.3390/pathogens10091124/s1, Table S1: Available genomic data for Trypanosomatidae.
